# Description of *Trichophoromyia ruifreitasi*, a new phlebotomine species (Diptera, Psychodidae) from Acre State, Brazilian Amazon

**DOI:** 10.3897/zookeys.526.6128

**Published:** 2015-10-08

**Authors:** Arley Faria José de Oliveira, Carolina Bioni Garcia Teles, Jansen Fernandes Medeiros, Luís Marcelo Aranha Camargo, Felipe Arley Costa Pessoa

**Affiliations:** 1 Centro de Pesquisa Leônidas e Maria Deane, Fundação Oswaldo Cruz, Rua Terezina, 476, Adrianópolis, Manaus, Amazonas, Brasil; 2 Fundação Oswaldo Cruz Rondônia - Fiocruz Rondônia, Rua da Beira, 7671, BR 364, Km 3,5, Bairro Lagoa, Porto Velho, Rondônia, Brasil; 3 Instituto de Ciências Biomédicas 5, Universidade de São Paulo (USP), Rua Francisco Prestes, 1234, Setor II, Monte Negro, Rondônia, Brasil; 4 Departamento de Medicina, Faculdade São Lucas, Rua Alexandre Guimarães, 1927, Areal, Porto Velho, Rondônia, Brasil

**Keywords:** Sand fly, taxonomy, Neotropical region, leishmaniasis, Psychodidae, Phlebotominae

## Abstract

*Trichophoromyia ruifreitasi* **sp. n.** is described as a new species of sand fly from the genus *Trichophoromyia* Barretto. This description is supported with illustrations and photographs that detail the morphological characteristics of male specimens collected in the municipality of Assis Brasil, Acre State, Brazilian Amazon. This species is similar to *Trichophoromyia auraensis* (Mangabeira), but the two species can be easily differentiated by the distribution of setae on their parameres, and by the presence of a dorsal lobe in the parameres of the new species.

## Introduction

Phlebotomine sand flies are small, dipteran, hematophagous insects. They are vectors of etiological agents such as *Leishmania* Ross, a protozoan that causes leishmaniases (Young and Duncan 1994). These diseases occur throughout the world, and infection can result in mutilations and death. Sand fly diversity is higher in the Amazon basin than it is in other biomes (Barret et al. 1996, Alves et al. 2012).

The genus *Trichophoromyia* Barretto is of medical importance because some species are involved in the life cycle of Leishmania (Viannia) lainsoni Silveira, Shaw, Braga and Ishikawa, and Leishmania (Viannia) braziliensis Vianna (Silveira et al. 1991, Martinez et al. 2001, Valdivia et al. 2012, Pereira Junior et al. 2015). To date, 41 species have been described of this diverse genus (Ladeia-Andrade et al. 2014, Fernandez et al. 2015). Most descriptions are based on male specimens, because the females of this genus are morphologically similar in most cases.

A study of phlebotomine sand fly diversity was undertaken in the region where Brazil borders Peru and Bolivia. A list of collected species was previously presented in Teles et al. (2013). These authors reported that *Trichophoromyia auraensis* (Mangabeira) is a known vector in that area (Valdivia et al. 2012; Araújo-Pereira et al. 2014). After reexamining the sand flies collected and identified as *Trichophoromyia auraensis*, it was discovered that the specimens belonged to a similar, but distinct species. The present paper describes this new species based on male specimens.

## Material and methods

Forest fragments were sampled in the municipality of Assis Brasil, located approximately 330 km south west of Rio Branco, Acre, Brazil, on the east bank of the Acre river, bordering Bolivia and Peru. Sand flies were captured between November 2009 and October 2010, using CDC light traps placed approximately 100 meters from domestic habitats. Details of the collection methods can be found in Teles et al. (2013). Insects were individually slide-mounted in synthetic Canada balsam. Specimens were identified and measured using a Zeiss microscope calibrated with a micrometer scale, and specimens were drawn using a camera lucida. All measurements are in micrometers (µm); measurements of the holotype are followed in parentheses by the measurement range of the paratypes, and the number of specimens observed. Morphological characteristics are also illustrated by photomicrographs that were made using a Leica DM 1000 optical microscope coupled to a JVC - 3 CCD digital camera and a computer imaging system.

Nomenclature and morphological terminology is according to Galati (2003).

## Taxonomy

### Trichophoromyia ruifreitasi sp. n.

Taxon classificationAnimaliaDipteraPsychodidae

http://zoobank.org/AFA99FEB-EDC6-4E1E-B46B-D346F16BD027

Figs 1 , 2 , 3

#### Type-material and depository.

Holotype male and 7 paratype males collected using CDC light traps in Assis Brasil, São Francisco road, 10°56'29"S 69°34'01"W, 5 -11.XII.2009, coll. L.M.A. Camargo. The holotype and paratypes are deposited in the entomological collection of the National Institute of Amazonian Research (INPA), and some paratypes are deposited at the Leônidas and Maria Deane Institute.

#### Diagnosis.

The new species is included in the genus *Trichophoromyia* due the male characters, the fifth palpomere slightly longer to the third, genitalia longer than or equal to the thorax, gonostyli with four spines (Santos et al. 2014). The new species is distinct from the others members of *Trichophoromyia* due the subtriangular paramere with a discrete dorsal lobe, and approximately 30 long, recurved setae distributed in the lobe, digital area after the dorsal proximal lobe is around 2× longer than it is broad, without distinct setose.

#### Description.

Male (n = 8) Holotype (male) small, measuring approximately 2040 (2000–2080, n = 8) from thorax to the end of the gonostylus. Head, thorax and abdomen brown, contrasting markedly with lower pleura and femora; paratergite, upper anepisternum, anepimeron and metepisternon pale.

**Figure 1.**
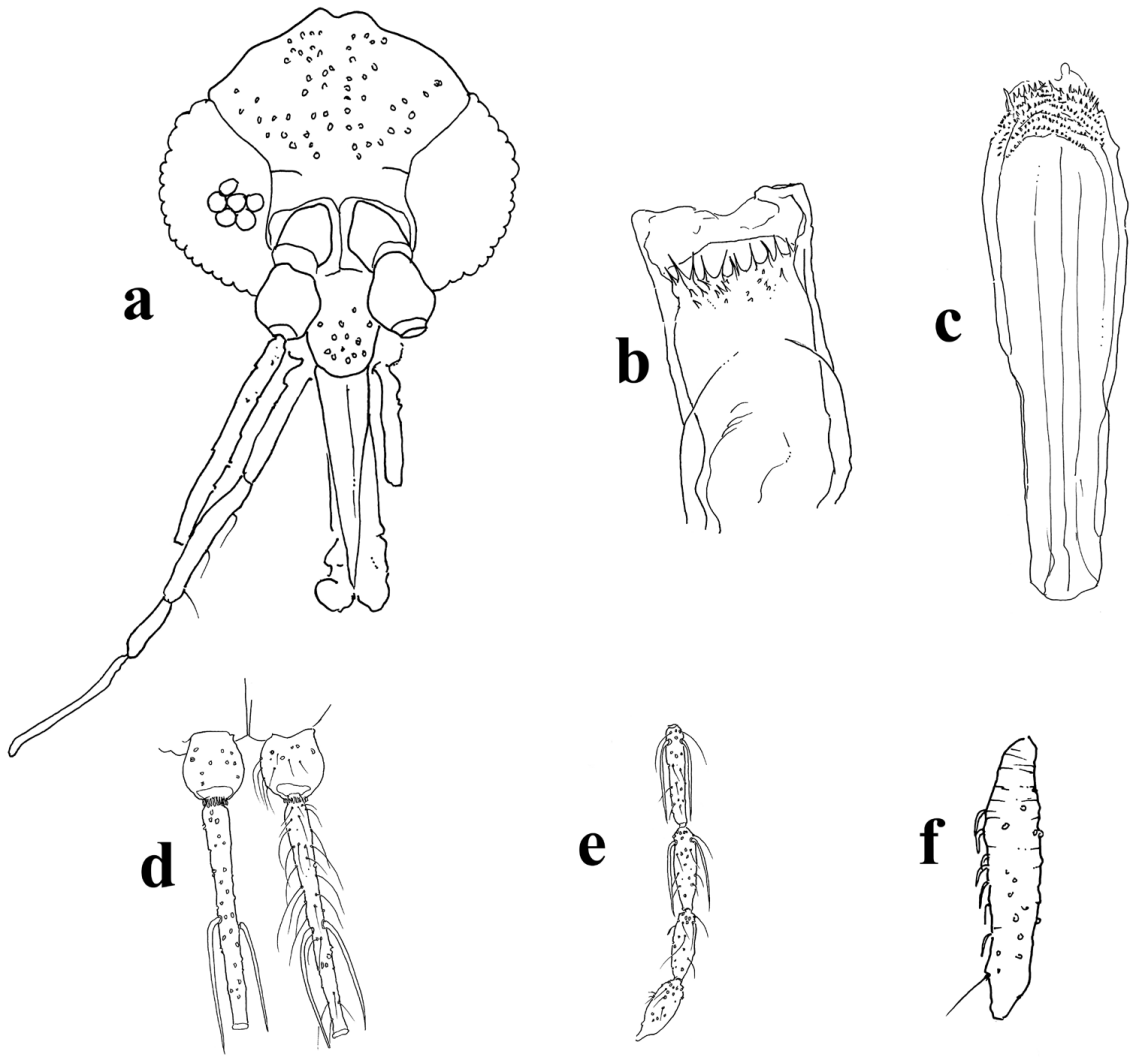
**A–F** *Trichophoromyia ruifreitasi* sp. n. **A** head, dorsal view **B** cibarium, dorsal view **C** pharynx, dorsal view **D–E** part of antenna, showing ascoids, dorsal view **F** palpomere III, dorsal view.

*Head* length 340 (325–340; n = 8) from post-occiput to clypeus apex, and maximum width 325 (300–325; n = 8). Eyes measuring 190 (190–195; n = 8) long by 100 (90–105; n = 8) wide, with incomplete interocular suture. Interocular distance 120 (105–120; n = 8) and ommatidia with a diameter of 18 (16–18; n = 8); interocular distance six times greater than the diameter of the ommatidia (Fig. 1a). Clypeus 101 (93–101; n = 8) long. Cibarium (Fig. 1b) with eight to ten acute posterior teeth equally spaced and clearly visible with a 40x objective; chitinous arc complete, pigmented spot weakly marked. Pharynx (Fig. 1c) 162 (160–173; n = 8) long, posterior third armed with transverse rows of denticles arranged in eight pairs and teeth clearly visible in immersion. Labrum-epipharynx 213 (200–216; n = 8) long. Antenna with simple, elongated ascoids (Fig. 1d) inserted nearly at the same level on antennomere AIII, reaching or exceeding the base of subsequent antennomeres, and present on all antennmoreres except XV and XVI (Fig. 1e). Length of antennomeres: AIII = 224 (213–224; n = 8), AIV = 125 (120–128; n = 8), AV = 122 (117–128; n = 8), AXV = 64 (64–69; n = 8) and AXVI = 56 (56–66; n = 8). Antennal formula = AIII–AXIV.2, AXV–AXVI.0. Palpus 445 (415–445; n = 8) long. Palpomeres: P1 = 35 (35–40; n = 8), P2 = 90 (80–90; n = 8), P3 = 130 (110–130; n = 8), P4 = 55 (50–60; n = 8), P5 = 135 (130–145; n = 7). Palpal formula: 1:4:2:3:5. Newstead’s spines distributed solely along the median inner face of palpomere III (Fig. 1f). Labial suture united in furca.

**Figure 2.**
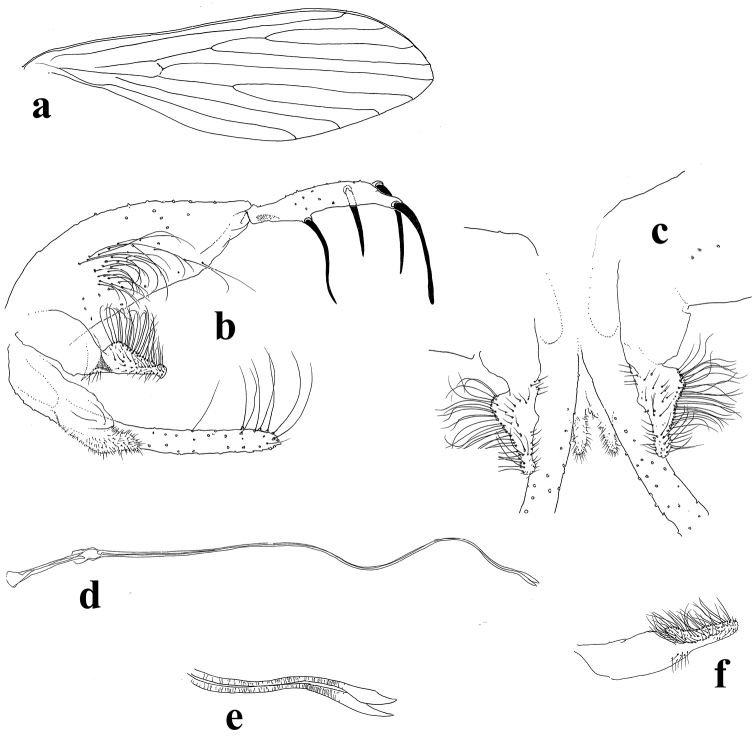
**A–F** *Trichophoromyia ruifreitasi* sp. n. **A** wing **B** lateral view of genitalia **C** dorsal view of parameres **D–E** genital filaments **F** paramere of *Trichophoromyia auraensis*, lateral view.

*Thorax* length 500 (480–580; n = 8) from anterior margin of pronotum to posterior margin of metanotum. Ventrocervical sensillae absent. Anepisternum with upper bristles 10 (10–13; n = 8) long, and lower bristles 5 (4–6; n = 8) long. Wing (Fig. 2a): length 1900 (1880–1900; n = 8) from insertion point to apex; maximum width 580 (580–600; n = 8). Venation: R5 = 1160 (1160–1222; n = 8) long; alpha = 520 (520–580; n = 8); beta = 260 (240–280; n = 8); delta = 340 (340–420; n = 8); gamma = 240 (220–240; n = 8); pi = 200 (200–220; n = 8); alpha twice the length of beta. Length of femora, tibiae, basitarsi and tarsomeres of fore, mid and hind legs: Fore: femora = 780 (720–800; n = 8); tibiae = 980 (940–1060; n = 8); basitarsi= 600 (580–620; n = 8); tarsomeres: I = 260 (260; n = 8), II = 180 (160–180; n = 8), III = 140 (140; n = 8), IV = 100 (100; n = 8). Mid: femora = 720 (680–740; n = 8); tibiae = 1220 (1140–1240; n = 8); basitarsi = 720 (680–720; n = 8), tarsomeres: I = 280 (280–300; n = 8), II = 180 (180–260; n = 8), III = 160 (140–160; n = 8), IV = 100 (100; n = 8). Hind: femora = 820 (780–840; n = 8); tibiae = 1400 (1320–1480; n = 8); basitarsi = 800 (740–820; n = 8); tarsomeres: I = 300 (260–300; n = 8), II = 200 (180–200; n = 8), III = 160 (160–180; n = 8), IV = 100 (100; n = 8). Hind femora without spines.

**Figure 3.**
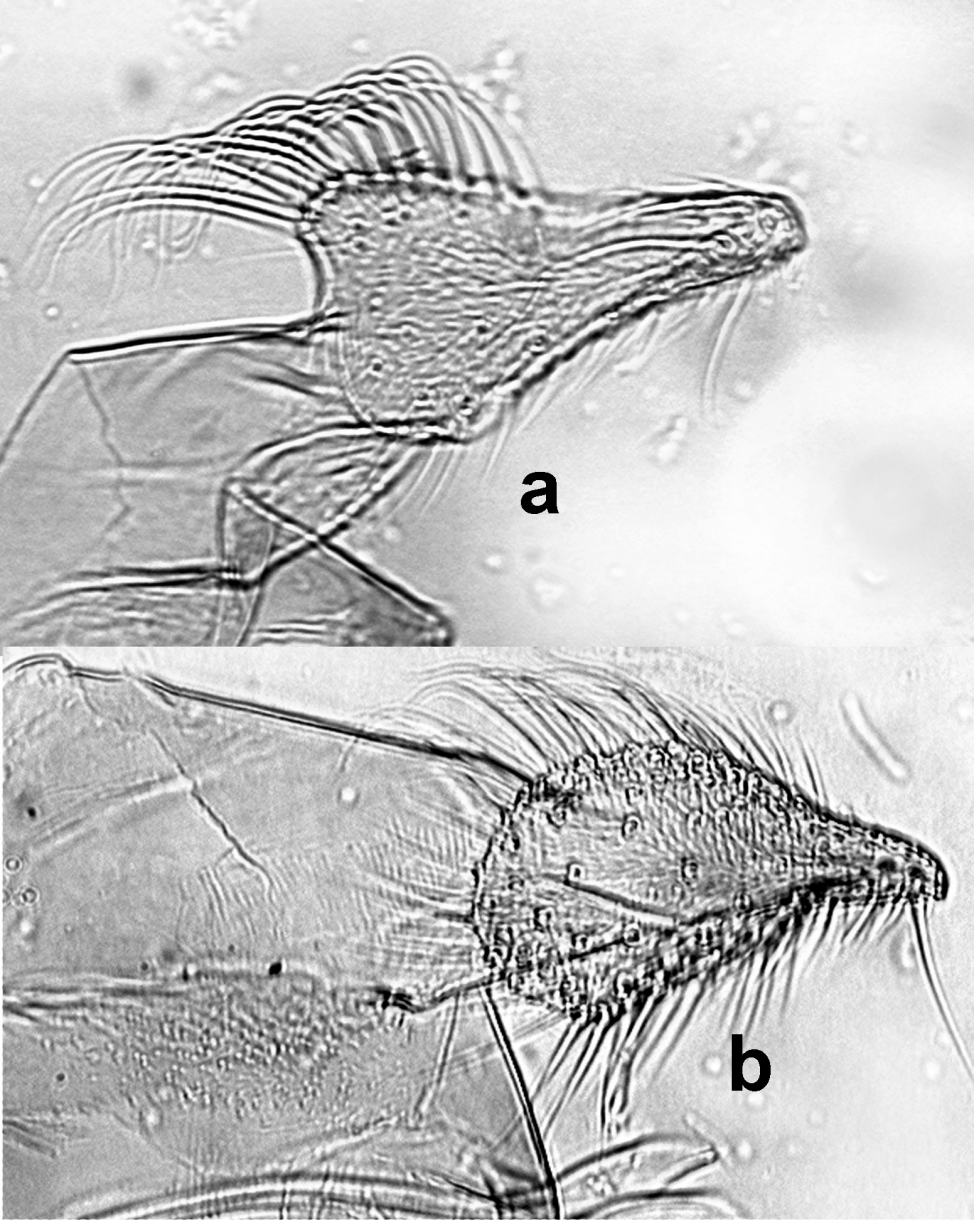
Lateral view of paramere of **A** *Trichophoromyia ruifreitasi* sp. n. **B** *Trichophoromyia auraensis*.

*Abdomen* length 2010 (1960–2110; n = 8) from first tergite to gonostylus apex. Genitalia (Fig. 2b): Gonostylus 185 (180–190; n = 8) long and 30 (30; n = 8) wide, presenting four strong spines distributed as follows: one apical, one subapical, one external implanted just below the subapical spine and equidistant from the apical and subapical spines, and one internal at the distal end of the gonostylus basal third; sub-terminal setae absent. Gonocoxite 320 (300–320; n = 8) long; maximum width 110 (80–120; n = 8), ornamented in the median area with a sparse group of approximately 30 bristles, some thin and long on the distal portion of the gonocoxite, but much shorter on the basal portion. Paramere (Figs 2b–c, 3a) 210 (200–210; n = 8) long, and 40 (35–45) wide, simple, subtriangular, proximal half part with a convex dorsal lobe, that is recovered with 28–30 long setae recurved at the apex; some setae running along the dorsal margin narrow at the rounded end of paramere, approximately ten (10) setae; apical margin with 4–5 much thicker setae. Proximal portion of paramere with a discrete translucid ventral lobe. Aedeagus conical and pigmented. Lateral lobe 350 (350–360; n = 8) long, cylindrical, not inflated, with a group of long slender setae that run along the back of the apex and spread throughout the distal half. Genital pump 180 (170–180; n = 8) long, and genital piston 150 (140–150; n = 8) long (Fig. 2d–e). Genital filaments (Fig. 2d–e) long and narrow with a striated surface; 900 (860–900; n = 8) long, thus approximately 5× longer than the pump. Apex of the filaments broad-bladed in shape and slightly recurved.

#### Etymology.

*Trichophoromyia ruifreitasi* is named in honor of our friend, mentor and fellow-researcher, Rui Alves de Freitas, who has made an immense and unparalleled contribution to the taxonomy of these small flies in Amazonas State.

#### Female.

Unknown.

## Discussion

*Trichophoromyia ruifreitasi* sp. n. and two other species of its genus share the same type locality. These species have distinct parameres: *Trichophoromyia auraensis* has a paramere (Figs 2f and 3b) that is completely covered with long setae, lacks a dorsal lobe, and is digitiform in the apical half. *Trichophoromyia ruifreitasi* has a subtriangular paramere with a discrete dorsal lobe, and approximately 30 long, recurved setae. *Trichophoromyia melloi* (Causey and Damasceno) has a paramere with a very pronounced dorsal lobe in the tip, with setae present solely within the apical region.

Two new *Trichophoromyia* species have recently been described in the Amazon basin: *Trichophoromyia nautaensis* in Loreto State, Peru, described by Fernandez, Lopez, Roldan and Requena; and, *Trichophoromyia adelsonsouzai* in Pará State, described by Santos, Silva, Barata, Andrade and Galati. Both species have parameres with dorsal lobes; however, in *Trichophoromyia nautaensis* the lobe is located in the median part of the paramere, while in *Trichophoromyia adelsonsouzai* the paramere has a relatively broad hump, exhibiting dorsal curvature in the apical region (Fernandez et al. 2015; Santos et al. 2014).

In comparison with other known species from the genus *Trichophoromyia*, the parameres of the new species, *Trichophoromyia napoensis* and *Trichophoromyia* sp.1 of Araracuara are similar. The digital area after the dorsal proximal lobe is approximately twice as long as it is broad in the new species compared with the other two species. However, *Trichophoromyia napoensis* is distinct from the others in that it possesses 2-3 long recurved setae at paramere apex, and a tuft of setae concentrated at the tip of dorsal lobe (Young and Duncan 1994). The parameres of *Trichophoromyia ruifreitasi* and *Trichophoromyia* sp.1 of Araracuara are covered by setae. *Trichophoromyia* sp.1 of Araracuara species possesses 4-6 long setae near its lateral ventral margin of the paramere apex (Young and Duncan 1994). Additionally, *Trichophoromyia* sp.1 of Araracuara also possesses other setae that are smaller than the width of the dorsal lobe, while *Trichophoromyia ruifreitasi* possess long setae that are distributed in the dorsal lobe. Santos et al. (2014) recently gave a brief review of the genus *Trichophoromyia*, and described *Trichophoromyia adelsonsouzai*, differentiating between the majority of species in the genus, except by the *Trichophoromyia napoensis* and *Trichophoromyia* sp.1 of Araracuara, not included in their analysis, and more closely related with the paramere of *Trichophoromyia ruifreitasi*. The new species described here raises the number of *Trichophoromyia* species worldwide to 42, and 21 in Brazil.

## Supplementary Material

XML Treatment for Trichophoromyia ruifreitasi

## References

[B1] Alves VR Freitas RA Santos FL Oliveira AFJ Barrett TV Shimabukuro PHF (2012) Sand flies (Diptera, Psychodidae, Phlebotominae) from Central Amazonia and four new records for the Amazonas state, Brazil. Revista Brasileira de Entomologia 56: 220–227. doi: 10.1590/S0085-56262012005000020

[B2] Araujo-Pereira T Fuzari AA Andrade Filho JD Pita-Pereira D Britto C Brazil RP (2014) Sand fly fauna (Diptera: Psychodidae: Phlebotominae) in na área of leishmaniasis transmission in the municipality of Rio Branco, state of Acre, Brazil. Parasites & Vectors 7: . doi: 10.1186/1756-3305-7-360 10.1186/1756-3305-7-360 PMC4141082 25103985

[B3] Barrett TV Freitas RA Albuquerque MIC Guerrero JCH (1996) Report on a Collection of *Lutzomyia* Sand Flies (Diptera: Psychodidae) from the Middle Solimões (Amazonas, Brazil). Memórias do Instituto Oswaldo Cruz 91: 27–35. doi: 10.1590/S0074-02761996000100005

[B4] Fernandez R Lopez V Cardenas R Requena E (2015) Description of Lutzomyia (Trichophoromyia) nautaensis n. sp. (Diptera: Psychodidae) from the Peruvian Amazon Basin. Journal of Medical Entomology, 1–4. doi: 10.1093/jme/tjv057 26335468 10.1093/jme/tjv057 PMC4592350

[B5] Galati EAB (2003) Morfologia e Taxonomia. Classificação de Phlebotominae. In: Rangel EF Lainson R (Eds) Flebotomíneos do Brasil. Fiocruz, Rio de Janeiro, 23–51.

[B6] Ladeia-Andrade S Fé NF Sanguinette CC Andrade-Filho JD (2014) Description of *Trichophoromyia uniniensis*, a new phlebotomine species (Diptera: Psychodidae: Phlebotominae) of Amazonas State, Brazil. Parasites & Vectors 7: . doi: 10.1186/1756-3305-7-400 10.1186/1756-3305-7-400 PMC4156613 25168121

[B7] Martinez E Le Pont F Mollinedo S Cupolillo E (2001) A first case of cutaneous leishmaniasis due to Leishmania (Viannia) lainsoni in Bolivia. Transactions of the Royal Society of Tropical Medicine and Hygiene 95: 375–377. doi: 10.1016/S0035-9203(01)90185-3 11579876 10.1016/s0035-9203(01)90185-3

[B8] Pereira Júnior AM Teles CBG Santos APA Rodrigues MS Marialva EF Pessoa FAC Medeiros JF (2015) Ecological aspects and molecular detection of *Leishmania* DNA Ross (Kinetoplastida: Trypanosomatidae) in phlebotomine sandflies (Diptera: Psychodidae) in terra firme and várzea environments in the Middle Solimões Region, Amazonas State, Brazil. Parasites & Vectors 8: . doi: 10.1186/s13071-015-0789-2 10.1186/s13071-015-0789-2 PMC4378226 25889808

[B9] Santos TV Silva FMM Barata IR Andrade AJ Galati EAB (2014) A new species of phlebotomine, *Trichophoromyia adelsonsouzai* (Diptera: Psychodidae) of Brazilian Amazonia. Memórias do Instituto Oswaldo Cruz 109: 140–147. doi: 10.1590/0074-0276130159 24141964 10.1590/0074-0276130159 PMC4015257

[B10] Silveira FT Souza AAA Lainson R Shaw JJ Braga RR Ishikawa EEA (1991) Cutaneous leishmaniasis in the Amazon region: natural infection of the sandfly *Lutzomyia ubiquitalis* (Psychodidae: Phlebotominae) by Leishmania (Viannia) lainsoni in Pará State, Brasil. Memórias do Instituto Oswaldo Cruz 86: 127–130. doi: 10.1590/S0074-02761991000100021 1842393 10.1590/s0074-02761991000100021

[B11] Teles CBG Freitas RA Oliveira AFJ Ogawa GM Araújo EAC Medeiros JF Pessoa FAC Camargo LMA (2013) Description of a new phlebotomine species (Diptera: Psychodidae, Phlebotominae) and new records of sand flies from the State of Acre, northern Brazil. Zootaxa 3609: 085–090. doi: 10.1590/0037-868216062013 10.11646/zootaxa.3609.1.6 24699574

[B12] Valdivia HO De Los Santos MB Fernandez R Baldeviano GC Zorrilla VO Vera H Lucas CM Edgel KA Lescano AG Mundal KD Graf PCF (2012) Natural *Leishmania* Infection of *Lutzomyia auraensis* in Madre de Dios, Peru, Detected by a Fluorescence Resonance Energy Transfer–Based Real-Time Polymerase Chain Reaction. The American Society of Tropical Medicine and Hygiene 87: 511–517. doi: 10.4269/ajtmh.2012.11-0708 10.4269/ajtmh.2012.11-0708 PMC3435357 22802444

[B13] Young DG Duncan MA (1994) Guide to the identification and geographic distribution of *Lutzomyia* sand flies in Mexico, the West Indies, Central and South America (Diptera: Psychodidae). Memoirs of the American Entomological Institute, Florida, United States of America, 881 pp.

